# Atherogenic index of plasma, lipid accumulation and visceral adiposity in metabolic syndrome patients

**DOI:** 10.6026/973206300181109

**Published:** 2022-11-30

**Authors:** Sabarinathan M, Deepak Rajan DS, Ananthi N, Madhan Krishnan

**Affiliations:** 1 Department of Biochemistry, Saveetha Medical College and Hospital, Saveetha Institute of Medical and Technical Sciences (SIMATS), Thandalam - 602015, Chennai, Tamilnadu, India; 2 Faculty of Allied Health Sciences, Chettinad Hospital and Research Institute, Chettinad Academy of Research and Education, Kelambakkam-603103, Tamilnadu, India

**Keywords:** Metabolic syndrome, Diabetes Mellitus, atherogenic index, adiposity index

## Abstract

Metabolic syndrome is a cluster of various clinical and biochemical abnormalities, needs early diagnosis and treatment to reduce morbidity and mortality. The present study is designed to compare Atherogenic Index of Plasma, Lipid Accumulation Product and Visceral Adiposity Index with metabolic syndrome components in patients with metabolic syndrome. The study comprises of 150 metabolic syndrome patients and 150 age and sex matched healthy controls of both genders in the age group of 20 - 65 years. Atherogenic Index of Plasma, lipid accumulation and Visceral Adiposity Index product index were calculated for all participants. Pearson Correlation was used to compareatherogenic Index of plasma, lipid accumulation and Visceral Adiposity Index product between cases and controls. The receiver operating characteristic curve (ROC) was used to compare the area under the ROC curve (AUC) of Atherogenic Index of Plasma, lipid accumulation and Visceral Adiposity Index product with metabolic syndrome. The comparisons between the BMI, WC, Atherogenic Index of Plasma ,lipid accumulation and Visceral Adiposity Index product were significantly higher in metabolic syndrome cases (p<0.001).Although the entire index were independently associated with Mets, AIP showed the highest area under the curve (0.954, 95% CI 0.929 0.978,p value p<0.0001) in identifying metabolic syndrome.

## Background:

Metabolic syndrome (MetS) is considered nowadays as an emerging public health issue due to its increased mortality and multiple morbidities across the globe [1]. It is a grouping of cardiovascular risk factors like type 2 diabetes mellitus (DM), obesity, dyslipidemia, and high blood pressure. Compared to individuals without the syndrome, patients with MetS have doubled the risk of cardiovascular diseases (CVD) and a higher incidence of developing type 2 diabetic mellitus [2]. MetS prevalence is alarmingly high and rising in both developing and developed nations [3]. Metabolic syndrome is becoming more common not only in the United States and Europe, but also in Asian countries such as China, India, and South Korea [4]. MetS prevalence ranges from 10% to as high as 84% worldwide, depending on geography, urban/rural environment, patient demographics (gender, age, race, and ethnicity), and definition [5]. The incidence of MetS differs between urban and rural populations in India. In urban Indians, the prevalence of MetS ranges from 19% to 45.3%, while in rural Indians, the prevalence rate was reported to range from 9.2% to 26.6% [6]. It could be generally caused on by a sedentary lifestyle, excessive food consumption, and the resulting abdominal obesity [4]. The Visceral Adiposity Index (VAI) is gender-specific, derived from simple anthropometric measures (BMI and WC) along with serum triglycerides and HDL cholesterol values [7]. It is effectively used to identify visceral fat distribution, IR, and elevated cardio metabolic risk [8]. Lipid Accumulation Product is an inexpensive tool for measuring lipid accumulation [9]. It is estimated using Waist circumference and serum triglyceride levels. There is a Strong correlation between the LAP index and MetS in the general population [7]. It was also found to be a simple, accurate, and stronger predictor of IR and MetS. It predicts cardiovascular disease with greater sensitivity and specificity than BMI and waist circumference [9]. Atherogenic index of plasma (AIP) is an unconventional lipid ratio that shows the logarithm of the molar ratio of TGs to HDL-C [10]. It is a measure of plasma atherogenicity due to a positive correlation with lipoprotein particle size, cholesterol esterification rates, and remnant lipoproteinemia. AIP not only accurately reflects the relationship between protective and atherogenic lipoproteins, but it is also a potent predictor of atherosclerosis and coronary heart disease [11]. Hence, the present study is designed to compare atherogen icindex of plasma, lipid accumulation product and visceral adiposity index with metabolic syndrome components in patients with metabolic syndrome and healthy controls.

## Materials and Methods:

This case control study conducted in the Clinical Biochemistry Laboratory at Saveetha Medical College and Hospital after obtaining approval from the Institutional Ethics committee. The study comprises of 150 metabolic syndrome patients and 150 age and sex matched healthy controls of both genders in the age group of 20 - 65 years. Subjects were chosen from patients who are attending the Out Patient department of Saveetha Medical College and Hospital. The written informed consent was obtained from the participants and they have been thoroughly explained about this study.

## Inclusion criteria:

## According to NCEP ATP III guidelines, three or more of the following criteria must be present to deem as having metabolic syndrome:

Waist circumference more than 102 cm for males, more than 88 cm for females

Blood pressure ≥ 130/85 mm Hg

Fasting blood glucose ≥ 110 mg/dl

HDL less than 40 mg/dl for males; less than 50 mg/dl for females

Plasma triglycerides ≥ 150 mg/dl

## Exclusion criteria:

Subjects Having Type I diabetes

 Renal diseases

 Hepatic diseases

 Cancer

 Thyroid dysfunction

 Hematological disorders

Anthropometric measurements such as body height, weight and waist circumference were taken and body mass index was calculated. Biochemical parameters such as Fasting plasma Glucose, Serum Triglycerides, Total Cholesterol and HDL cholesterol were estimated within 3 hours of sample collection in both metabolic syndrome patients and healthy controls using Vitros 5600 integrated fully automated analyzer.

## The indices were calculated as follows:

AIP = log (TGL/HDL)

 VAI (Male) = (WC/ (39.68 + (1.88 x BMI)) x (TGL/1.03) x (1.31/HDL)

VAI (Female) = (WC/ (36.58 + (1.89 x BMI)) x (TGL/0.81) x (1.52/HDL)

 LAP (Male) = (WC - 65) x TGL

 LAP (Female) = (WC - 58) x TGL

## Statistical analysis:

Statistical analysis of data was performed using Statistical Package for the Social Science (SPSS) version 26.0. Descriptive statistics wasexpressed as Mean and standard deviation were calculated. An Independent samples t-test was performed to compare the means between metabolic syndrome patients and controls. Association between inflammatory markers and Mets components were done by using Pearson's correlation test. A p-value of less than0.05 was taken as statistically significant. Diagnostic Performance of the inflammatory markers was analysed by using Area under Curve (AUC) obtained by constructing Receiver Operating Characteristic (ROC) Curves.

## Results:

The present study has included 150 metabolic syndrome patients and 150 controls in the age group of 20 - 65 years. Table 1 shows the Sex distribution between cases and controls. Table 2 shows the comparison of anthropometric and biochemical parameters between cases and controls. Table 3 shows the correlation of LAP, VAI & AIP with Metabolic Syndrome Components such as WC, SBP, DBP, FPG, TGL and HDL by using Pearson Correlation. Correlation of LAP with WC, SBP, FPG, TGL and HDL showed a highly statistical significant correlation with the (r value - 0.436, 0.222, 0.216, -0.193, 0.792) and (p value - < 0.0001, 0.006, 0.008, 0.018, < 0.0001). LAP with DBP did not found a statistically significant correlation with the r value (0.132) and p value (0.108). **Correlation is significant at the 0.01 level. *Correlation is significant at the 0.05 level. Correlation of VAI with TGL and HDL showed a highly statistical significant correlation with the (r value - 0.774, 0.759) and (p value - < 0.0001, < 0.0001).VAI with WC, SBP, DBP and FPG did not found a statistically significant correlation with the r value (-0.118, 0.123, 0.030,-0.069) and p value (0.151, 0.135, 0.715, 0.399). Correlation of AIP with WC, TGL and HDL showed a highly statistical significant correlation with the (r value - 0.235, 0.851,-0.825) and (p value - < 0.0001, < 0.0001, < 0.0001). AIP with SBP, DBP and FPG did not found a statistically significant correlation with the r value (0.097, 0.016,-0.090) and p value (0.238, 0.845, 0.275).

## Discussion:

Diabetes mellitus is a word that refers to a group of illnesses or syndromes that are characterised by uncontrolled hyper glycemia. It's a carbohydrate, lipid, and protein metabolism condition characterized by insulin secretion insufficiency (relative or total) and different degrees of insulin resistance. It is a metabolic illness characterized by aberrant carbohydrate, protein, and lipid metabolism as a result of elevated blood insulin levels or enhanced insulin sensitivity in the target tissues [12]. Obesity, ageing, genetic predisposition, and physical inactivity are all linked to obesity. Diabetes mellitus, a global public health concern, is presently spreading like an epidemic over the world[13].According to the research (IDF 2019), 463 million people worldwide have diabetes, with that figure expected to climb to 578 million by 2030 and 700 million by 2045 [14].CVDs are the leading cause of death worldwide. As a result, CVDs are expected to cause 32% of all deaths worldwide in 2019. In low- and middle-income countries, more than 75% of CVD-related deaths occur. Atherogenic index of plasma (AIP) and visceral adiposity index (VAI) are relatively new indicators for predicting non-communicable diseases (NCDs) [15]. MetS involve interrelated factors such as abdominal obesity, insulin resistance, hyperglycemia, hypertension, and dyslipidemia (low high-density lipoprotein and increased triglyceride). Individuals are defined as they possess metabolic syndrome in case of the presence of at least 3 of the 5 traits mentioned and are insulin resistant.[16].An important CVD risk factor, atherogenic dyslipidemia is characterized by a rise in TG and low-density lipoprotein cholesterol (LDL-C) levels and a fall in HDL-C levels in the blood [17].In the present study Correlation of LAP with WC, SBP, FPG, TGL and HDL showed a highly statistical significant correlation with the (r value - 0.436, 0.222, 0.216, -0.193, 0.792) and (p value - < 0.0001, 0.006, 0.008, 0.018, < 0.0001). LAP with DBP did not found a statistically significant correlation with the r value (0.132) and p value (0.108).According to some populations, the atherogenic index of plasma (AIP), which is the logarithmic conversion of the TG to HDL-C ratio, is a reliable indicator of atherosclerosis and CVDs. One of the most sensitive markers of CVDs, AIP is correlated with the lipoprotein particle size of LDL-C, HDL-C, and very-low-density lipoprotein (VLDL) [18].Additionally, the accuracy and dependability of VAI for predicting T2DM, hypertension, and MetS have been demonstrated by predictive power of VAI using receiver operating characteristic (ROC) curve analysis [19]. The current ROC curve analysis, the AIP showed the highest area under the curve (0.954, 95% CI 0.929 0.978, p value ≤ 0.0001) followed by the LAP (0.898, 95% CI 0.862 - 0.934, p value ≤0.0001) VAI (0.931, 95% CI 0.901 -0.961, p value ≤0.0001) in identifying metabolic syndrome (Table 4; Figure 1-see PDF).

## Conclusion:

 The comparisons between atherogenic index of plasma, lipid accumulation product and visceral adiposity index with metabolic syndrome components in patients with MetS and healthy controls is the metabolic syndrome cases had significantly higher levels of BMI, WC, atherogenic Index of Plasma, lipid accumulation, and Visceral Adiposity Index product (p 0.001). AIP had the highest area under the curve (0.954, 95% CI 0.929 0.978, p value 0.0001) in identifying metabolic syndrome even though the entire index was independently associated with Mets.
